# Commentary: Synergistic treatment of sodium propionate and Sishen Pill for diarrhea mice with kidney-yang deficiency syndrome

**DOI:** 10.3389/fcimb.2025.1646463

**Published:** 2025-08-13

**Authors:** Leyao Fang, Na Deng

**Affiliations:** School of Traditional Chinese Medicine, Hunan University of Chinese Medicine, Changsha, China

**Keywords:** kidney-yang deficiency syndrome, intestinal microbiota, short-chain fatty acid, gut-kidney axis, Sishen Pill

## Introduction

Diarrhea is clinically characterized by increased stool frequency and loose or watery stool consistency. Kidney-Yang deficiency syndrome is one of the most common traditional Chinese medicine (TCM) patterns associated with diarrhea, and is characterized by a prolonged disease course and a high tendency for recurrence. Studies have shown that diarrhea with kidney-Yang deficiency syndrome is frequently accompanied by gut microbiota dysbiosis and altered microbial metabolites (Li et al., 2022). Short-chain fatty acids (SCFAs), such as acetate, propionate, and butyrate, are essential metabolites produced by gut bacteria. These metabolites play key roles in modulating intestinal barrier integrity and host immune responses (Howard et al., 2022). Among them, propionate has been shown to exert anti-inflammatory effects (Yuenyongviwat et al., 2021). In addition, it has been reported to alleviate diarrheal symptoms by reducing intestinal motility (Song et al., 2022). Diarrheal mice with kidney-Yang deficiency syndrome have significantly lower propionate levels, which are associated with a gut microbiota imbalance (Li et al., 2022). Sishen Pill, a classical TCM formula, is widely used in the treatment of diarrhea with kidney-Yang deficiency syndrome. Recent evidence indicates that Sishen Pill has the potential to enhance host intestinal function by restoring the balance of gut microbiota (Zhu et al., 2023). Propionate reduces intestinal inflammation, while Sishen Pill modulates gut microbial composition; both effectively alleviate diarrheal symptoms through mechanisms closely linked to the gut microbiota. Exploring the potential synergistic effects of sodium propionate and Sishen Pill in treating diarrhea with kidney-Yang deficiency syndrome may enhance therapeutic efficacy. Additionally, determining their optimal combination ratio could provide valuable experimental evidence to guide the optimization of traditional Chinese herbal formulas and the development of novel pharmaceutical preparations.

## General comments

A recent study entitled “Synergistic treatment of sodium propionate and Sishen Pill for diarrhea mice with kidney-yang deficiency syndrome” was published in Frontiers in Cellular and Infection Microbiology. Guo et al. investigated the therapeutic benefits of the concurrent administration of Sishen Pill and sodium propionate on diarrheal mice with kidney-Yang deficiency syndrome, specifically highlighting their regulatory influences on gut microbiota composition, microbial activity, and enzymatic function. This study established a mouse model of diarrhea with kidney-Yang deficiency syndrome using a combination of adenine and *Folium sennae*. Different dosages of sodium propionate were empirically selected and administered alongside Sishen Pill to assess their impact on general condition, diarrheal symptoms, immune organ indices, gut microbiota abundance, intestinal microbial activity, and enzymatic activities. The results demonstrated that the combination of 75% Sishen Pill with 60 mg/kg sodium propionate exerted the most favorable therapeutic effect. This regimen significantly alleviated the clinical aspects of diarrhea with kidney-yang deficiency syndrome by effectively inhibiting *Escherichia coli* overgrowth, promoting *Bifidobacterium* restoration, and increasing intestinal enzymatic activities. The authors concluded that the overall therapeutic efficacy of the combination therapy was superior to that of Sishen Pill or sodium propionate alone.

This study presents several notable strengths. First, the intervention groups were thoughtfully designed to explore the potential synergy between Sishen Pill and sodium propionate across different dose gradients. Second, the evaluation system is comprehensive, encompassing general symptoms, immunological organ indices, and both structural and functional aspects of the gut microbiota, thus enabling a multidimensional assessment of therapeutic efficacy. In addition, the study is grounded in contemporary microbial ecology theory and utilizes gut microbiota-derived metabolites as a starting point, offering a novel perspective for understanding the mechanisms of TCM herbal formulations. Finally, the investigation of the synergistic effects between Sishen Pill and sodium propionate provides valuable insights for the development of compound TCM formulations.

However, several limitations should be noted in the current study. While the modeling aligns with the principle of integrating disease and TCM syndrome. However, it largely relies on subjective interpretation of symptoms rather than standardized pathological or molecular markers, potentially limiting reproducibility. Although the study reported a synergistic effect between sodium propionate and Sishen Pill, it lacked formal quantitative analyses to statistically validate this interaction. Without such analyses, the claim of synergy remains descriptive and requires further validation through rigorous pharmacological modeling. Future research should adopt established approaches, such as combination index analysis, to distinguish between synergistic, additive, and antagonistic effects across different dose combinations. Such methods would enhance the pharmacological foundation of combination therapies involving TCM and microbiota-derived metabolites. Moreover, the mechanisms underlying the observed synergy remain unclear. Propionate may enhance the efficacy of Sishen Pill by improving the absorption and bioavailability of its active components or by modulating endogenous propionate levels and function. Previous research has shown that SCFAs can enhance intestinal tight junction protein expression and reinforce mucosal barrier function, thereby reducing inflammation and oxidative stress (Liu et al., 2021). Given that diarrhea with kidney Yang deficiency causes damage to both intestinal and renal tissues, which are interconnected via the gut-kidney axis, propionate may exert its therapeutic effects through this pathway (Li et al., 2024). However, this hypothesis requires experimental validation. Future investigations could examine histopathological changes in colonic and renal tissues; markers of barrier integrity; renal function parameters; and the expression of key signaling molecules and inflammatory cytokines. Free fatty acid receptor (FFAR) 2 and FFAR3 are key SCFA receptors mediating the anti-inflammatory effects of acetate and propionate, and their decreased expression has been implicated in various diseases (Fusco et al., 2023; Zhang D. et al., 2023). Propionate primarily signals through FFAR3, and it is plausible that Sishen Pill enhances this effect by upregulating FFAR2 and FFAR3 expression. To confirm their role in the synergistic mechanism, future studies may evaluate FFAR2/3 expression in colonic tissues using immunohistochemistry, and perform receptor inhibition experiments to determine their involvement in mediating the combined therapeutic effects.

In addition, while Guo et al. investigated the effects of combining sodium propionate and Sishen Pill on gut microbiota composition, microbial activity, and enzymatic function in diarrheal mice with kidney-Yang deficiency syndrome, further metagenomic research is warranted. In this instance, the study did not provide an in-depth investigation of propionate’s regulatory effects on key bacterial taxa, nor did it clarify whether Sishen Pill modulates propionate-producing bacteria in a targeted manner. SCFAs, as essential metabolites derived from gut microbiota, are directly connected with human health and disease conditions (Hays et al., 2024). Acetate, propionate, and butyrate are produced by different bacterial taxa, which regulate SCFA synthesis through distinct metabolic pathways (Sittipo et al., 2025). SCFA levels also alter with aging in gut microbial populations (Tsukuda et al., 2021). During infancy, acetate predominates and is primarily synthesized by *Bifidobacteria*. After weaning, propionate levels increase and are produced via three major metabolic pathways: the succinate pathway (e.g., *Prevotella*, *Veillonella*), the acrylate pathway (e.g., *Coprococcus*), and the propanediol pathway (e.g., *Roseburia*, *Blautia*). Butyrate is primarily produced by bacteria from the Lachnospiraceae and Ruminococcaceae groups (Fusco et al., 2023). Recent investigations indicate substantial variations in gut microbiota composition across different TCM disorders (Shen et al., 2025), with kidney-Yang deficiency syndrome demonstrating age-related microbial characteristics. The gut microbiota composition and propionate levels in diarrheal mice with kidney-Yang deficiency differ markedly from those in healthy controls. To gain deeper insight into the molecular function of propionate in this disease, high-throughput 16S rRNA gene sequencing could be used to assess gut microbiota changes after propionate administration and identify key taxa involved in propionate metabolism. Similarly, examining changes in the abundance of propionate-producing bacteria following Sishen Pill treatment may help elucidate its modulatory effects on these microbial communities.

Finally, this study addressed the dose gradient of sodium propionate and its potential synergistic interaction with Sishen Pill, thereby providing preliminary evidence for a novel combinatory strategy integrating TCM formulas with microbiota-derived metabolites for treating diarrhea with kidney-Yang deficiency syndrome. However, the selection of sodium propionate doses appears empirical and lacks pharmacokinetic or pharmacodynamic validation. The absence of either systematic dose-response modeling or absorption, distribution, metabolism, and excretion (ADME) analyses raises concerns about the scientific rigor and translational relevance of the dosing strategy. Future studies should prioritize evidence-based pharmacological evaluations to optimize dosing and enhance clinical applicability. The incorporation of probiotics in TCM fermentation offers additional advantages, including enhanced pharmacological efficacy, reduced toxicity, and the production of novel bioactive compounds (Zhang X. et al., 2023). The integration of TCM formulations with probiotics has attracted increasing attention, suggesting a promising direction for synergistic applications (Liu et al., 2025). Collectively, the current findings and previous research lay a robust foundation for future mechanistic studies and product development, underscoring their promising translational value and clinical applicability. In addition, critical parameters such as chemical stability and batch consistency of the combined formulation were not extensively assessed, which may offer hurdles for future development. Future research should expand on these findings by conducting comprehensive pharmacokinetic profiling, including ADME analyses, to inform rational dose selection. Importantly, interindividual variability in gut microbiota composition and its impact on therapeutic response should be further explored to support the development of personalized combinatory treatment approaches. Such integrative approaches will not only optimize therapeutic efficacy but also expand the clinical indications of this compound therapy to include chronic diarrhea, irritable bowel syndrome, and inflammatory bowel disease, thereby contributing to the modernization and internationalization of TCM-based interventions.

## Discussion

We sincerely acknowledge Guo et al.’s contribution to investigating the combined use of Sishen Pill and sodium propionate in treating diarrhea with kidney-Yang deficiency syndrome. Their findings provide preliminary insights into how TCM formulations may interact with gut microbiota-derived metabolites, contributing to the modernization of TCM through the lens of microbial ecology. Nevertheless, the study’s methodological and experimental design aspects warrant further refinement to enhance rigor and reproducibility. While the results suggest a potential synergistic effect between Sishen Pill and sodium propionate, the underlying mechanisms remain unclear and require more systematic and mechanistic investigation. The gut-kidney axis, which reflects the bidirectional interaction between intestinal and renal systems, offers a promising framework for exploring how this combinatory treatment exerts therapeutic effects in the context of kidney-Yang deficiency. The integration of multi-omics technologies, including high-throughput sequencing and metabolomics, could aid in identifying key microbial taxa and clarifying the complex interplay between TCM components, gut microbiota, and microbial metabolites. In parallel, the development of standardized medicinal formulations and personalized treatment strategies will be critical for advancing clinical translation and maximizing the therapeutic potential of this approach.
